# Isotropic Contact Properties in Monolayer GeAs Field-Effect Transistors

**DOI:** 10.3390/molecules28237806

**Published:** 2023-11-27

**Authors:** Weiqi Song, Haosong Liu, Feihu Zou, Yize Niu, Yue Zhao, Yao Cong, Yuanyuan Pan, Qiang Li

**Affiliations:** 1 College of Physics, Center for Marine Observation and Communications, Qingdao University, Qingdao 266071, China; 2021020281@qdu.edu.cn (W.S.); 2021020280@qdu.edu.cn (F.Z.); 2022025400@qdu.edu.cn (Y.N.); zhaoyue69@qdu.edu.cn (Y.Z.); 2 State Key Laboratory of Heavy Oil Processing, Institute of New Energy, College of Chemical Engineering, China University of Petroleum (East China), Qingdao 266580, China; lhs_upc@163.com (H.L.); cong_y1217@163.com (Y.C.)

**Keywords:** monolayer GeAs, quantum transport simulations, Schottky barrier, field-effect transistors

## Abstract

Owing to the tunable bandgap and high thermodynamic stability, anisotropic monolayer (ML) GeAs have arisen as an attractive candidate for electronic and optoelectronic applications. The contact properties of ML GeAs with 2D metal (graphene, Ti_2_CF_2_, V_2_CF_2_, and Ti_3_C_2_O_2_) and Cu electrodes are explored along two principal axes in field-effect transistors (FET) by employing ab initio electronic structure calculations and quantum transport simulations. Weak van der Waals interactions are found between ML GeAs and the 2D metal electrodes with the band structure of ML GeAs kept the same, while there is a strong interaction between ML GeAs and the Cu metal electrode, resulting in the obvious hybridization of the band structure. Isotropic contact properties are seen along the two principal directions. *P*-type lateral Schottky contacts are established in ML GeAs FETs with Ti_3_C_2_O_2_, graphene, and Ti_2_CF_2_ metals, with a hole Schottky barrier height (SBH) of 0.12 (0.20), 0.15 (0.11), and 0.29 (0.21) eV along the armchair (zigzag) direction, respectively, and an *n*-type lateral Schottky contact is established with the Cu electrode with an electron SBH of 0.64 (0.57) eV. Surprisingly, ML GeAs forms ideal *p*-type Ohmic contacts with the V_2_CF_2_ electrode. The results provide a theoretical foundation for comprehending the interactions between ML GeAs and metals, as well as for designing high-performance ML GeAs FETs.

## 1. Introduction

Benefitting from the atomic thickness, promising good gate control and smooth surfaces without dangling bonds facilitating carrier transport, two-dimensional (2D) semiconductors, such as silicene [1], transition metal dichalcogenides (TMDs) [2], Bi_2_O_2_Se [3], and black phosphorene (BP) [4,5], have attracted considerable research interest and show promise in nano-electronic and optoelectronic applications. Different from the isotropic 2D semiconductors with extremely high crystal symmetry and in-plane isotropy, the anisotropic 2D semiconductors with low symmetry have unique electrical, mechanical, and optical properties, which opens up the possible application in the angle-dependent devices resulting from an extra degree of freedom in the plane [6,7,8,9]. A family of low-symmetry anisotropic 2D semiconductors, few-layer, and monolayer (ML) IV-V semiconductors (e.g., GeP, GeAs, SiP, and SiAs) have recently been fabricated and possess excellent structural stability [10,11,12]. Additionally, due to their high in-plane anisotropy, they exhibit tunable optical [13,14,15,16,17], electronic [11,18,19,20], and thermoelectric properties [21,22], resulting in a wide field of applications involving polarization sensors, artificial synaptic devices, and integrated digital inverters [23,24]. 

The 2D GeAs, as the most famous member of the novel 2D IV-V semiconductors, has attracted extensive attention. It has been fabricated by liquid phase and micromechanical exfoliation with good stability in ambient conditions [13,14,25,26], and the predicted similar exfoliation energy of GeAs (0.37 J/m^2^) and graphite (0.32 J/m^2^) allows ML GeAs to be easily fabricated from bulk GeAs as well [27,28,29,30]. Additionally, few-layer GeAs transistors with a back-gate geometry have a maximum hole field-effect mobility of 100 cm^2^ V^−1^ s^−1^ and a high on–off ratio of up to 10^5^ [14,26,31]. An anisotropic mobility ratio of 4.6 is reached by few-layer GeAs, surpassing that of other 2D anisotropic semiconductors, such as black phosphorene (1.5) and ReS_2_ (3.1) [25,32,33]. These excellent properties make ML GeAs a promising 2D semiconductor candidate for field-effect transistors (FETs). Since contacting with metals is inevitable in 2D semiconductor applications, metal−semiconductor contact properties are critical for the practical application of ML GeAs devices. Recently, a large Schottky barrier height (SBH) of 0.40~0.49 eV was found between the multilayer 2D GeAs, and Au/Cr/GeAs junctions have been found in the broadband Schottky photodiode [34]. Theoretically, the electron transport properties of graphene and 2D IV-V semiconductors were studied, and graphene contacts inject additional charge carriers into the ML GeAs channel, thereby enhancing its electron transport properties [35]. However, the SBH and electron transport properties between the ML GeAs and other bulk metals are still lacking. The performance of 2D-layer metals as electrodes is better than that of bulk metals, while there are few studies on their contact with ML GeAs. Therefore, it is necessary to investigate the ideal metal contact to achieve outstanding performance for ML GeAs FETs.

In this work, we employ ab initio electronic structure calculations and quantum transport simulations to examine the interactions between ML GeAs and metal electrodes (graphene, Ti_2_CF_2_, V_2_CF_2_, Ti_3_C_2_O_2_, and Cu) in the FETs. ML GeAs maintains its band structure when it contacts with 2D metal, while the bandgap of ML GeAs disappears, and metallization occurs when it contacts with the Cu electrode. Identical types of Schottky barriers are present along the two principal directions. By using quantum transport simulations, ML GeAs forms vertical Ohmic barriers with V_2_CF_2_, Ti_3_C_2_O_2_, and Cu electrodes, while it forms *p*-type vertical Schottky barriers with graphene and Ti_2_CF_2_ electrodes in ML GeAs FETs. Furthermore, ML GeAs forms *p*-type lateral Schottky barriers with graphene, Ti_2_CF_2_, and Ti_3_C_2_O_2_, while it forms the *n*-type lateral Schottky barrier when Cu is used as an electrode. In particular, ML GeAs forms an ideal *p*-type Ohmic contact with the V_2_CF_2_ electrode. In the ML GeAs/metal contact system, the pinning factor of −0.23 is obtained, resulting in a strong Fermi level pinning (FLP). These findings reveal a fundamental comprehension of barriers in ML GeAs FETs and a guide for the design of promising ML GeAs devices.

## 2. Results and Discussion 

### 2.1. Interface and Device Models

**Figure 1:**
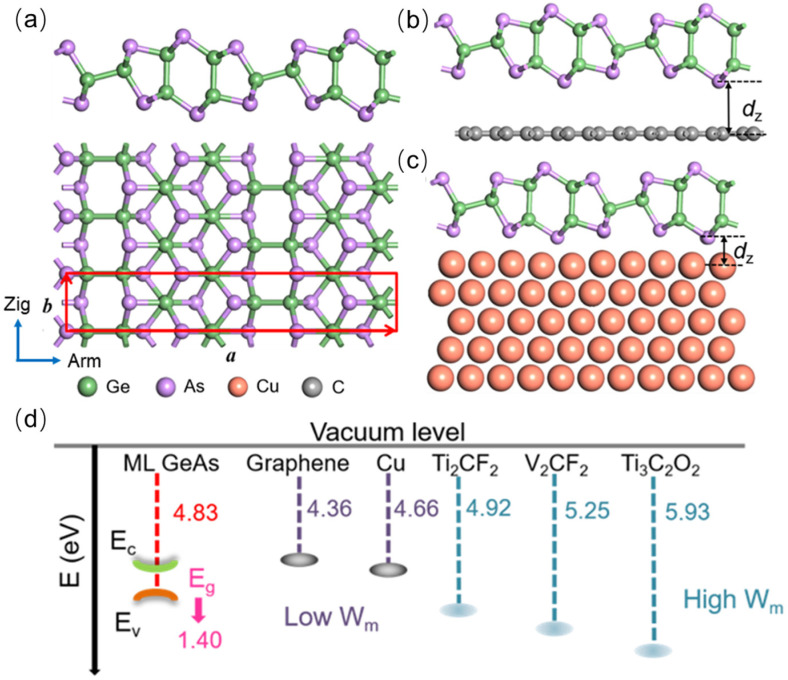
(**a**) Side and top views of the optimized ML GeAs. The red rhombus indicates the unit cell of ML GeAs. Arm and Zig represent the armchair and zigzag directions, respectively. (**b**) Side view of ML GeAs contacting with 2D metal and (**c**) bulk metal. (**d**) Band alignments between ML GeAs and metals. *E*_c_, *E*_v_, and *E*_g_ represent the conduction band minimum, the valence band maximum, and the bandgap of ML GeAs, respectively.

Top contact is the commonly used configuration in 2D semiconductor FETs, and most of the 2D GeAs device configuration is the top contact in experiments [18,26,35]. Therefore, we chose the top contact to study the interfacial properties in the ML GeAs FETs. As described in Figure 1b,c, the metal surface of the bulk electrode was simulated by five layers of metal atoms, while one layer was chosen to simulate the metal surface of 2D metals, in which one side of the metal surface absorbs ML GeAs. The top three layers of bulk metals mainly interact with ML GeAs; thus, we fixed the two bottom layers. The lattice constants of the ML GeAs and independent metal electrodes are shown in Table S1. The plane of ML GeAs is the (001) surface of bulk GeAs. The lattice parameters of the selected electrodes were adjusted to those of ML GeAs, and the mismatches of the lattice parameters between ML GeAs and Cu, graphene, Ti_2_CF_2_, Ti_3_C_2_O_2_, and V_2_CF_2_ were 0.32%, 2.36%, 3.04%, 3.09%, and 3.18%, respectively [36,37]. The 5$\sqrt{3}$ × 3 unit cells of graphene and Cu (111) surfaces, as well as the 2$\sqrt{13}$ × $\sqrt{7}$ unit cells of Ti_3_C_2_O_2_, V_2_CF_2_, and Ti_2_CF_2_ surfaces, are compatible with the 1 × 2 unit cells of ML GeAs. The work function of these selected electrodes covers a wide range, from 4.36 eV to 5.93 eV in Figure 1d, which favors modification of the carrier polarity of ML GeAs FET. The calculated work functions of graphene and Cu were 4.36 eV and 4.66 eV, respectively, which are very close to the experimental values of 4.60 eV and 4.65 eV [38,39,40]. 

We built a two-probe ML GeAs FET to investigate their transport properties. The channel region and the left/right electrode region were comprised of pristine ML GeAs and the optimized ML GeAs/metal interfaces, respectively. The transport properties of ML GeAs FET were investigated using a 5~7 nm channel length along the armchair and zigzag directions because of the high in-plane anisotropy. The lengths of the left and right electrodes were semi-infinite.

### 2.2. Structure and Electronic Properties of the ML GeAs/Metal Interfaces

Figure 1a shows the optimized structure of ML GeAs, in which every As atom is bonded to three Ge atoms, and every Ge atom is bonded to three As atoms together with one additional Ge atom. After optimization, the lattice parameters of the ML GeAs were *a* = 22.25 Å and *b* = 3.82 Å, and this result is compatible with the earlier theoretical and experimental studies [12,29]. In addition, the structure of ML GeAs illustrates the high in-plane anisotropy along the armchair and zigzag directions, which leads to its excellent properties in serval fields [26]. Thus, the transport properties along the armchair and zigzag directions are studied below. 

**Figure 2:**
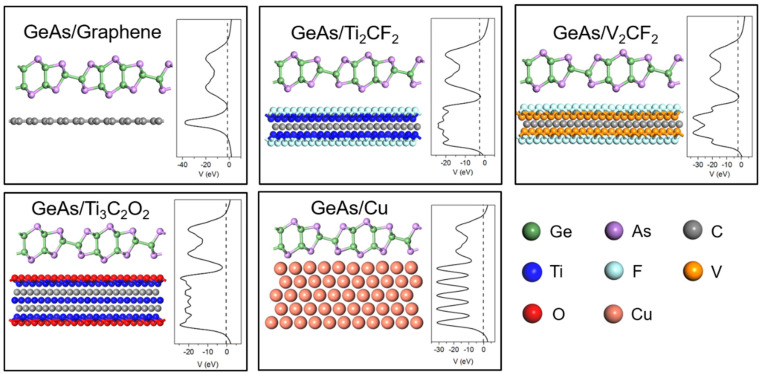
Side views of the optimized structures and the average electrostatic potential distribution in the planes normal to the interfaces of ML GeAs on graphene, Ti_2_CF_2_, V_2_CF_2_, Ti_3_C_2_O_2_, and Cu surfaces. The Fermi level is represented by the black dashed lines.

From Figure 2, the side views of the optimized ML GeAs/metal interfaces and the pristine structure of ML GeAs are basically preserved on all metal surfaces. The essential parameters of the ML GeAs/metal interfaces are indicated in Table 1. As presented in Figure 1b,c, the interlayer distance, *d*_z_, is the average vertical distance between the As atoms at the bottom of the ML GeAs and the top atoms of the metal layer. The interlayer distances between the ML GeAs and Cu, Ti_3_C_2_O_2_, V_2_CF_2_, Ti_2_CF_2_, and graphene were 2.04, 2.58, 2.63, 2.67, and 3.28 Å in the optimized interfaces, respectively. We also calculated the binding energy, *E*_b_, of the interfaces to further illustrate the interactions between them, which is defined in the equation below.

*E*_b_ = (*E*_metal_ + *E*_GeAs_ − *E*_GeAs/metal_)/*N*

where *E*_metal_, *E*_GeAs_, and *E*_GeAs/metal_ represent the relaxed energy of the free-standing metal surface, the free-standing ML GeAs, and the ML GeAs/metal contact system, respectively, and *N* is the number of Ge and As atoms closest to the metal surface in the calculated supercells. The binding energies, *E*_b_, of the ML GeAs/graphene, Ti_3_C_2_O_2_, V_2_CF_2_, Ti_2_CF_2_, and Cu systems were 0.39, 0.91, 0.97, 0.99, and 2.96 eV/atom, as listed in Table 1, respectively. Depending on the *E*_b_ and *d*_z_, there are two classes of interactions between ML GeAs and metals: weak bonding and strong bonding. The ML GeAs interactions with Ti_3_C_2_O_2_, V_2_CF_2_, Ti_2_CF_2_, and graphene established weak bonds with a larger interlayer distance of 2.5 Å < *d*_z_ < 3.3 Å and a smaller binding energy of *E*_b_ < 1 eV/atom. The sum of covalent radii of the As and metal atoms in the weak bonding was substantially more than the vertical interlayer distance; thus, ML GeAs and metals were connected by the vdW-type stack. The vdW interaction was also observed in the ML GeP/graphene heterostructure [10]. In particular, the ML GeAs/graphene interface formed the largest interlayer distance and the smallest binding energy, which means that the interaction between ML GeAs and graphene was the weakest, while the ML GeAs/Cu interface formed strong bonds with a smaller interlayer distance of *d*_z_ < 2.5 Å and a larger binding energy of *E*_b_ > 1 eV/atom.

**Figure 3:**
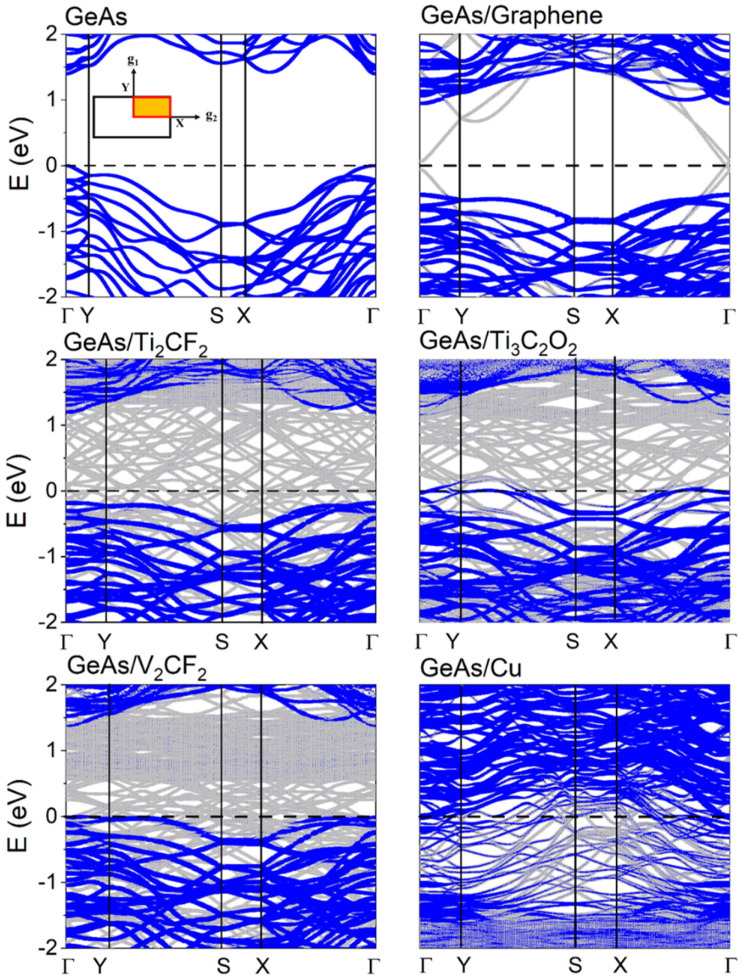
Band structures of ML GeAs and ML GeAs on graphene, Ti_2_CF_2_, V_2_CF_2_, Ti_3_C_2_O_2_, and Cu surfaces. Gray and blue lines represent the band structure of the interfacial systems and are projected to the ML GeAs in the interfacial systems, respectively. The line width is proportional to the weight. The Fermi level is set at zero energy and denoted by the black dashed line.

To further study the coupling of the ML GeAs with metals, the band structure is estimated in Figure 3, and the results are similar to previous studies. The valence band maximum (VBM) and the conduction band minimum (CBM) of ML GeAs, which are both positioned in the Γ point, showed that the semiconductor direct bandgap was 1.40 eV [26,29]. According to the formula $\left(\frac{1}{m*}=\frac{1}{{\text{ℏ}}^{2}}\frac{d^{2}E}{dk^{2}}\right)$, the effective mass, m*, of ML GeAs was obtained from the band structure. The electron effective masses were 0.34 *m*_e_ and 0.14 *m*_e_ (*m*_e_ is the mass of an electron) along the Γ→X and Γ→Y, respectively, while the hole effective masses were 0.28 *m*_e_ and 0.80 *m*_e_ along the Γ→X and Γ→Y, respectively. In comparison with ML BP, the electron effective masses were equal or even smaller [32], the effective mass of ML GeP was lower, and the anisotropic mobility was better; thus, ML GeAs had a substantially reduced effective mass [41]. Therefore, with a low effective mass and great effective mass anisotropy, ML GeAs exhibited excellent anisotropic mobility.

**Figure 4:**
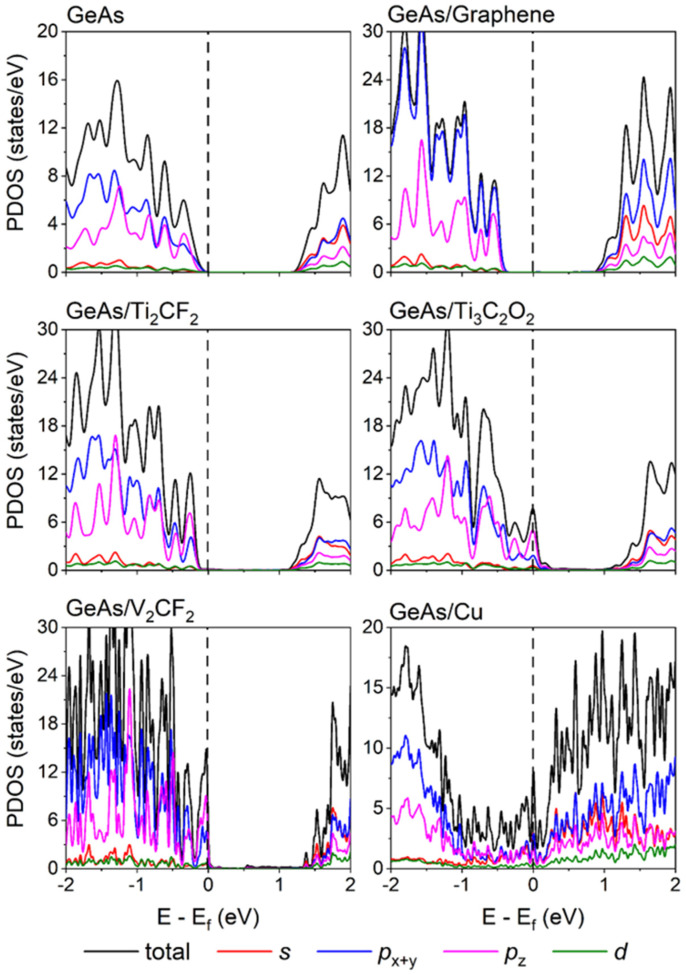
Partial density of states (PDOS) of the pristine ML GeAs and ML GeAs on graphene, Ti_2_CF_2_, V_2_CF_2_, Ti_3_C_2_O_2_, and Cu surfaces. The Fermi level is set at zero and denoted by the black dashed line.

Figure 3 displays the weak interaction between ML GeAs and graphene, while Ti_2_CF_2_, Ti_3_C_2_O_2_, and V_2_CF_2_ metals preserved the intrinsic band structures of ML GeAs. The bandgap of ML GeAs was also retained with the values of 1.19, 1.31, 1.33, and 1.41 eV for the ML GeAs with V_2_CF_2_, Ti_3_C_2_O_2_, Ti_2_CF_2_, and graphene metal contact systems, respectively. However, the Fermi level (*E*_F_) of ML GeAs shifted up/down owing to the charge transfer between ML GeAs and metals. It moved slightly upward when ML GeAs contacted graphene and Ti_2_CF_2_, while it moved downward when ML GeAs contacted V_2_CF_2_ and Ti_3_C_2_O_2_. The bandgap disappeared when ML GeAs contacted Cu, destroying the band structure of ML GeAs and leading to metallization with Cu. To gain an improved comprehension of the electron states of the contact systems, Figure 4 evaluates the partial density of states (PDOS) of these systems. The electron states of the pristine ML GeAs near the CBM were mostly the result of the hybridization of *p_x_*_+ *y*_ and *s* orbitals, while those near the VBM were mostly contributed by the *p* orbital. After ML GeAs contacted the metals, the bandgap of ML GeAs was well maintained with the weak bonding, and the electron state contribution close to the CBM and VBM was consistent with that in the pristine ML GeAs. The strong interaction between the interfaces caused a large number of DOS to appear in the bandgap of ML GeAs in the ML GeAs/Cu interface, together with the small interlayer distances of 2.04 Å and the large binding energy, *E*_b_, of 2.96 eV/atom in the ML GeAs/Cu interface, which indicates the formation of metallization of ML GeAs. 

### 2.3. Schottky Barriers in the ML GeAs FETs

**Figure 5:**
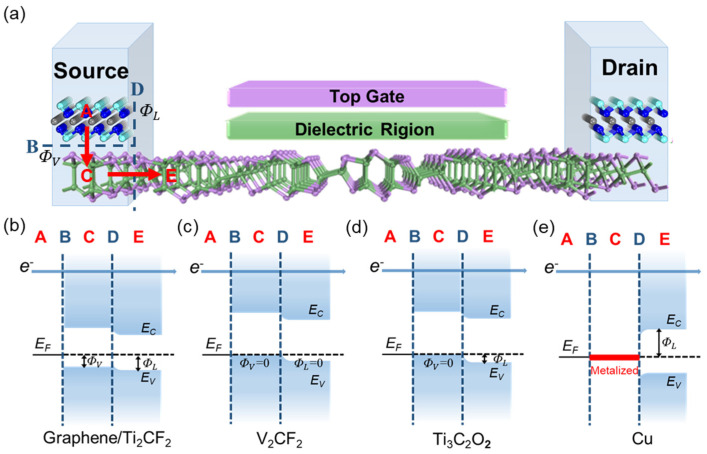
(**a**) Schematic diagram of ML GeAs FET. The red arrows represent the pathways of the carrier transfers from the electrode to the channel ML GeAs, and the blue dashed lines indicate two interfaces at ML GeAs FET, i.e., vertical interface B and lateral interface D. $\Phi_{V}$ and $\Phi_{L}$ represent the corresponding vertical and lateral SBH, respectively. (**b**–**e**) Four possible schematic diagram illustrations based on quantum transport calculations of ML GeAs FETs with graphene, Ti_2_CF_2_, V_2_CF_2_, Ti_3_C_2_O_2_, and Cu electrodes. *E*_C_ and *E*_V_ are the conduction and valence band edges of the ML GeAs, respectively. *E*_F_ denotes the Fermi level of the ML GeAs−electrode junctions.

To further examine the transport properties, Figure 5 presents a two-probe ML GeAs FET with a channel length of 5~7 nm. Two different interfaces will be passed through when carriers transfer from the electrode to the channel ML GeAs: one is interface B, which is in the vertical interface between ML GeAs and the electrode surfaces, and the other is interface D, which is in the lateral interface between the ML GeAs/metal contact systems and the channel ML GeAs. When carriers passed through the gap between the metal and the underlying ML GeAs, the tunneling barrier could be observed at interface B. Schottky barriers may additionally influence carriers at interfaces B and D. According to previous reports, the electrostatic potential barrier higher than *E*_F_ in interface systems is referred to as the tunneling barrier [42,43]. The tunneling barrier did not exist for the studied ML GeAs/metal interfaces because the electrostatic potential at those interfaces was below the *E*_F_, as illustrated in Figure 2. Thus, the tunneling probability was 100% at the ML GeAs/metal contacts.

#### 2.3.1. Vertical Schottky Barriers in the ML GeAs FETs

As one of the most important parameters of metal−semiconductor contact field effectors, SBH is an important factor that determines the carrier injection efficiency and resistance of devices. In the vertical direction in Figure 3, the band structures can be used to determine the electron/hole vertical Schottky barrier, $\Phi_{V}^{e/h}$, which is defined as the energy difference between the *E*_F_ of the contact systems and the identifiable CBM/VBM of the ML GeAs in the electrode. The band structures of pristine ML GeAs were well preserved when ML GeAs contacted the 2D metal electrodes because of the weak interaction between them. Obviously, ML GeAs/Ti_3_C_2_O_2_ and ML GeAs/V_2_CF_2_ systems observed *p*-type Ohmic contacts with the *E*_F_ below the VBM of ML GeAs, while ML GeAs/graphene and ML GeAs/Ti_2_CF_2_ systems observed *p*-type Schottky contacts with the hole SBH values of 0.45 eV and 0.19 eV, respectively. Particularly, the ML GeAs/Cu system observed the Ohmic contact owing to the band of ML GeAs being hybridized with the Cu electrode.

Furthermore, because the metal electrode interacts with the ML GeAs in the channel region, the vertical SBH calculated by the band structure calculation may be unreliable without taking the channel region into account [44]. In addition, the work function approximation is the commonly employed method to examine lateral SBH, which fails to recognize the coupling between the channel region and the metal electrode region [45,46,47]. Based on the FET model, quantum transport simulations are an increasingly trustworthy method for identifying both the vertical and lateral SBHs in 2D semiconductor FETs, which considers the interaction between the electrode and the channel region because these two regions form a whole [44,48,49]. Therefore, in the following parts, quantum transport simulations are investigated in depth to explore the SBH in the ML GeAs transistors. 

**Figure 6:**
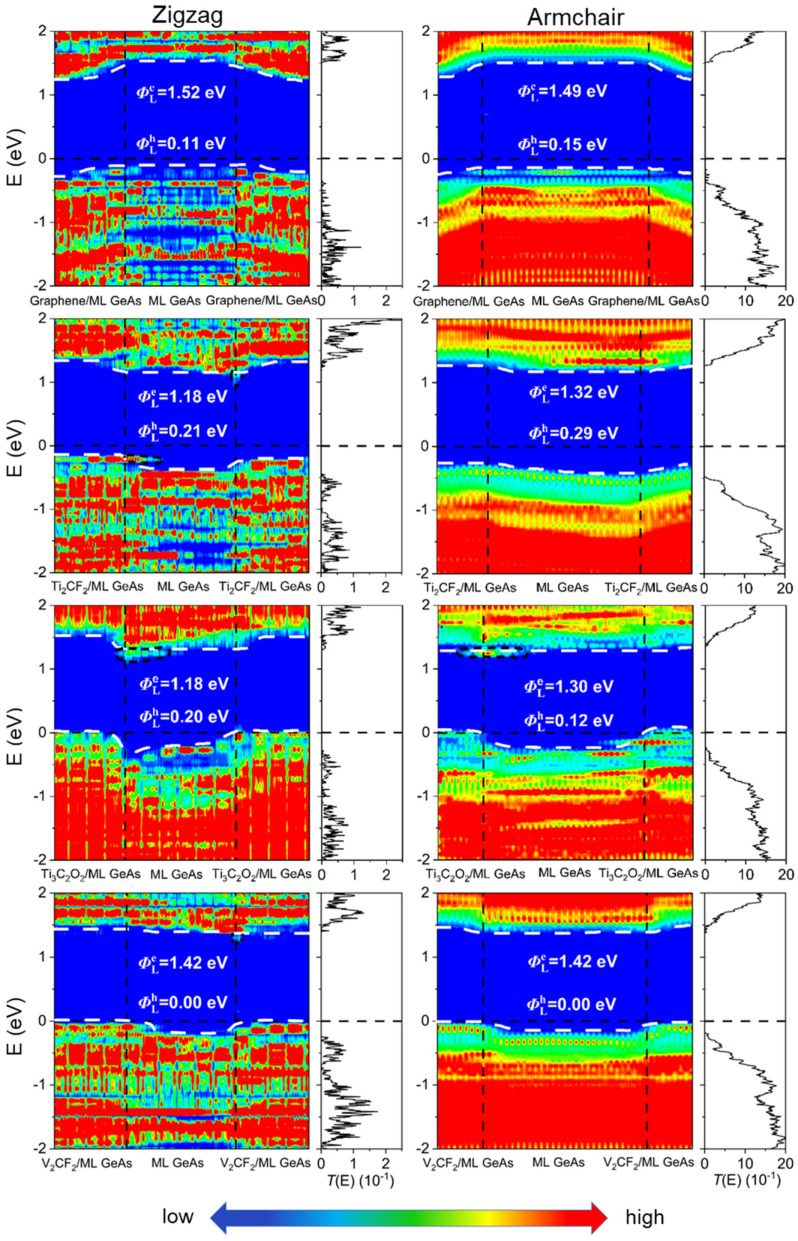
Zero-bias and zero-gate voltage LDDOS and transmission spectra of the ML GeAs FETs with graphene, Ti_2_CF_2_, Ti_3_C_2_O_2_, and V_2_CF_2_ electrodes. The Fermi level is set at zero. The left and right figures represent ML GeAs along the armchair and zigzag directions, respectively. The white dashed lines represent the VBM and CBM of ML GeAs. The interface states are represented by the black, short-dashed lines.

**Figure 7:**
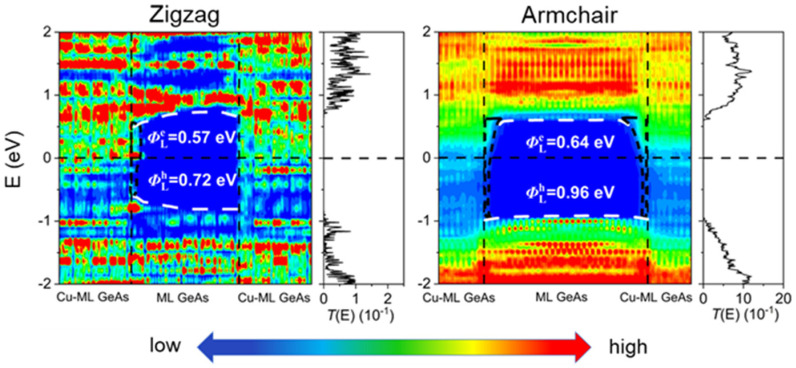
Zero-bias and zero-gate voltage LDDOS and transmission spectra of the ML GeAs FETs with Cu electrodes. The Fermi level is set at zero. The left and right figures represent ML GeAs along the armchair and zigzag directions, respectively. The white dashed lines represent the VBM and CBM of ML GeAs. The interface states are represented by the black, short-dashed lines.

According to quantum transport simulations, the local device density of states (LDDOS) and the transmission spectra of the ML GeAs FETs were obtained under zero-bias and zero-gate voltage, as depicted in Figure 6 and Figure 7. The ML GeAs/metal vertical contacts displayed isotropy and shared the same contact type in the zigzag and armchair directions. Compared with the result from the band structure calculation, the contact types of the ML GeAs/metal interface of the two methods were the same; that is, ML GeAs/graphene and ML GeAs/Ti_2_CF_2_ contact systems formed *p*-type Schottky contacts, whereas with Ti_3_C_2_O_2_, V_2_CF_2_, and Cu electrodes, ML GeAs formed *p*-type Ohmic contacts. In particular, in the electrode region, electronic states obviously appeared in the bandgap because of the metallization caused by the strong interaction between ML GeAs and Cu, thus forming a *p*-type vertical Ohmic barrier. However, quantum transport simulations of the $\Phi_{V}$ were calculated with the hole SBH values of 0.25 eV and 0.54 eV when ML GeAs contacted graphene and Ti_2_CF_2_ electrodes, which is different from that calculated by the electronic structure calculations with the hole SBH values of 0.45 eV and 0.19 eV, respectively.

#### 2.3.2. Lateral Schottky Barriers in the ML GeAs FETs

Meanwhile, based on the quantum transport simulations, we also studied the lateral SBH of the ML GeAs FETs with graphene, Ti_2_CF_2_, Ti_3_C_2_O_2_, V_2_CF_2_, and Cu electrodes. Along the armchair/zigzag directions, the lateral electron/hole Schottky barriers, $\Phi_{L}^{e,\;A/Z}\;$and${\;\Phi }_{L}^{h,\;A/Z}$, were assessed through the energy differences between the $E_{F}$ of the contact systems and the CBM/VBM of the ML channel GeAs at interface D:

$$\Phi_{L}^{e,\;A/Z}=E_{C}\;-\;E_{F}\;\mathrm{and}\;\Phi_{L}^{h,\;A/Z}=E_{F}\;-\;E_{V}$$

where *E*_C_ and *E*_V_ denote the values of CBM and VBM of the ML GeAs channel, respectively, and *E*_F_ is the Fermi level of the ML GeAs FETs. Similar to vertical SBH, ML GeAs/metal also showed isotropy for lateral SBH in both directions. ML GeAs with graphene, Ti_2_CF_2_, and Ti_3_C_2_O_2_ metals observed *p*-type Schottky carriers with the hole SBH values of 0.11 (0.15) eV, 0.21 (0.29) eV, and 0.20 (0.12) eV in the zigzag (armchair) direction, respectively, and formed a *p*-type Ohmic carrier in the ML GeAs/V_2_CF_2_ system with an $E_{F}$ below the VBM of the ML GeAs channel in both directions. When ML GeAs contacted Cu, it formed an *n*-type Schottky carrier with the electron SBH value of 0.57 (0.64) eV in the zigzag (armchair) direction, respectively. Meanwhile, there existed observable interfacial states at the ML GeAs/Cu interface, regarded as metal-induced gap states (MIGS). Therefore, the V_2_CF_2_ electrode contact with ML GeAs can form an ideal Ohmic carrier in both directions, making it a promising candidate electrode for the ML GeAs FET. The right sides of Figure 6 and Figure 7 depict the transmission spectra of the contact systems. ML GeAs with graphene, Ti_2_CF_2_, Ti_3_C_2_O_2_, and V_2_CF_2_ electrodes formed *p*-type carriers, and formed an *n*-type Schottky carrier in both directions in the ML GeAs/Cu contact system. The lateral SBH and polarity determined by transmission spectra broadly agreed with those obtained from the LDDOS.

### 2.4. Discussion

According to the SBHs determined by the quantum transport simulations, the isotropic ML GeAs/metal contacts were divided into four types in Figure 5b–e. As illustrated in Figure 5b, the ML GeAs/graphene and ML GeAs/Ti_2_CF_2_ contact systems observed *p*-type Schottky barriers in both the vertical (interface B) and the lateral directions (interface D), with the bandgap of ML GeAs maintained in both the electrode and channel regions. As illustrated in Figure 5c, owing to the $E_{F}$ being lower than the VBM of ML GeAs with the bandgap maintained, the ML GeAs/V_2_CF_2_ contact system observed a *p*-type Ohmic barrier in both directions, which can facilitate electron transport into the ML GeAs FET. As shown in Figure 5d, the ML GeAs/Ti_3_C_2_O_2_ contact system observed a *p*-type vertical Ohmic barrier and a *p*-type lateral Schottky barrier. In Figure 5e, due to the strong interaction leading to MIGS, significant metallization was observed along the vertical direction for the ML GeAs/Cu system, forming an *n*-type lateral Schottky barrier. The findings demonstrate that the desired Ohmic contact can be achieved by ML GeAs-based FETs with the V_2_CF_2_ electrode, which is advantageous to the construction of high-performance ML GeAs FETs. The smaller Schottky barriers always make ML GeAs FETs perform better. Therefore, the electrode priority for p-type ML GeAs FETs is V_2_CF_2_ > Ti_3_C_2_O_2_ > graphene > Ti_2_CF_2_. The V_2_CF_2_ electrode with Ohmic contacts is an ideal electrode for ML GeAs FETs. Further, the contact type could also guide the design of ML GeAs devices in experiments. 

**Figure 8:**
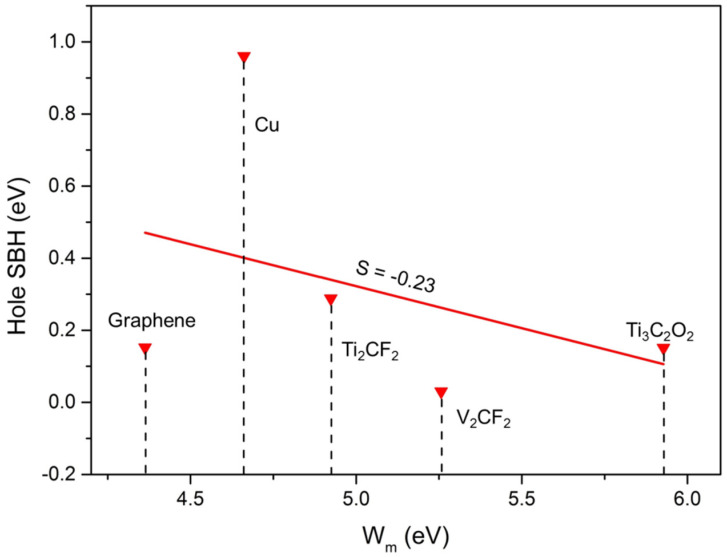
Relative hole SBHs of the ML GeAs/metal junctions vs. the work function values of metals. *S* is the pinning factor according to the Schottky−Mott rule.

FLP is always observed in 2D metal–semiconductor interfaces, which leads to uncontrollable high contact resistance and unmodulated semiconductor polarity, further resulting in a great challenge in developing reliable 2D semiconductor electronic devices [50]. The level of FLP is described by the pinning degree, *S*, which can be calculated by the formula: *S* = |d″*Φ*_L_″/d*W*_m_|, where “*Φ*_L_” represents the SBH of ML GeAs/metal junctions in the lateral direction, and Wm is the work function of the electrode. No FLP is indicated by the pinning factor *S* = ±1, while a strong FLP is indicated by *S* = 0. Figure 8 plots the relationship between the side hole SBH calculated through the work function and quantum transport calculation, which obtained *S* = −0.23. Therefore, the pinning factor was close to 0, indicating a relatively strong pinning effect. This significant pinning effect was also observed in the other 2D semiconductor FETs and is usually attributed to the presence of MIGS and defect states; for example, MoS_2_ (*S* = 0.27) and WSe_2_ (*S* = 0.32) [51,52,53]. The MIGS appeared in ML GeAs FETs at the lateral interfaces with Ti_2_CF_2_, Ti_3_C_2_O_2_, and Cu, as shown in Figure 6 and Figure 7, and no defects existed in these FETs. Thus, the strong FLP was mainly from the MIGS in ML GeAs FETs.

## 3. Computational Methods

Based on density functional theory (DFT), the ML GeAs/metal interfaces were obtained using the Vienna ab initio simulation package (VASP) Version 5.4.4 code to assess the geometry optimizations and the electronic properties [54,55]. All calculations were delivered under the Perdew–Burke–Ernzerhof (PBE) method, with the generalized gradient approximation (GGA) applying a projector-augmented wave (PAW) pseudopotential approach in order to discover the exchange-correlation functional [56,57]. A 450 eV cutoff energy was applied to the plane-wave basis set. On the other hand, the DFT-D3 method of Grimme with zero damping was employed to consider the vdW (van der Waals) correction for the weak interaction between the two adjacent layers [58]. The energy and residual force had a convergence threshold of less than 1 × 10^−5^ eV and 0.02 eV/Å, respectively. Using the Gamma-centered *k*-point grid density of 0.02 Å^−1^, the Brillouin zone was sampled [59]. The vacuum buffer layer was adjusted to more than 15 Å to meet the decoupling between its adjacent plates.

The Quantum Atomistix Toolkit (QuantumATK) Version P-2019.03 software package implemented the nonequilibrium Green’s function (NEGF) method coupled with DFT to compute the transport properties of ML GeAs transistors with different electrodes [60,61,62,63]. The linear combination of the atomic orbits (LCAO) basis set was used to examine the double-ξ polarization (DZP) form. The device sampled 8 × 1 × 1 and 8 × 1 × 50 Monkhorst–Pack *k*-point meshes for the channel and electrode regions, respectively [59]. The density mesh cutoff was 100 Hartree, and the temperature was set to 300 K. The following equation defines the transmission coefficient, $T\;(k_{y},\;E)$:

$$T(k_{y},\;E)=Tr[\Gamma_{L}(k_{y},\;E)G(k_{y},\;E)\Gamma_{R}(k_{y},\;E)G^{+}(k_{y},E)]$$

where *k_y_* is the *y* portion of the reduced wave vector, and $G(k_{y},\;E)$ ($G^{+}(k_{y},E)$) represents a retarded (advanced) Green function in the central region. $\Gamma_{L/R}=i(\;\sum_{L/R}^{r}-\;\sum_{L/R}^{a})$ represents the level broadening resulting from the left/right electrode in the form of self-energy, $\sum_{L/R}$. *L/R* stands for the left/right electrode. The essential Hamiltonian to explain the lead–device interaction is the electrode self-energy.

In the 2D FETs, the 2D semiconductors contacting the metals were substantially doped with carriers, shielding the electron–electron interaction. Thus, the GGA-PBE based on a single electron is a trustworthy approximation to evaluate the SBHs, which was also proven in previous work [48]. For instance, the calculated hole SBHs using the GGA-PBE method were 0.34, 0.19, and 0.20 eV for ML-, bilayer-, and tri-layer-phosphorene with the Ni electrode transistors, respectively [42,45,64], which are qualitatively consistent with the experimental results of 0.35, 0.23, and 0.21 eV, respectively [65].

## 4. Conclusions

Based on ab initio electronic structure calculations and quantum transport simulations, the contact properties of ML GeAs and metals (graphene, Ti_2_CF_2_, V_2_CF_2_, Ti_3_C_2_O_2_, and Cu) were investigated in the transistors. The coupling of ML GeAs with the Cu electrode led to metallization and the destruction of the band structure of the ML GeAs, while ML GeAs formed a vdW interaction with the other studied metals, with the band structure of ML GeAs being maintained. ML GeAs/metal interfaces showed isotropic properties with the same contact type and a similar Schottky barrier height along the armchair and zigzag directions. *P*-type lateral Schottky contacts were observed when graphene, Ti_2_CF_2_, and Ti_3_C_2_O_2_ were employed as electrodes, while an *n*-type lateral Schottky contact was formed when ML GeAs contacted the Cu electrode. In particular, the ML GeAs/V_2_CF_2_ contact system formed the ideal *p*-type Ohmic contact. The pinning factor calculated at the ML GeAs/metal interface was −0.23; thus, it had a strong FLP effect, which may be decreased by inserting a 2D material to achieve the ideal Ohmic contact. This study not only offers a comprehensive overview of the ML GeAs FET’s interfacial properties, but also identified the V_2_CF_2_ electrode as an ideal electrode for ML GeAs transistors.

## Figures and Tables

**Table 1:** Calculated interfacial properties of ML GeAs/metal interfaces. $\bar{\varepsilon }$ is the average absolute lattice constant mismatch between ML GeAs and metal electrodes. The equilibrium distance, *d*_z_, is the average distance of the ML GeAs and metal in the vertical direction. The binding energy, *E*_b_, is the energy per Ge or As atom to remove ML GeAs from the metal surface. *W*_m_ and *W* are the calculated work functions of the clean metal surface and the ML GeAs/metal systems, respectively. $\Phi_{L}^{e/h}$ is the transport SBH of the electron (hole) in the vertical direction. $\Phi_{L}^{e,\;A/Z}{(\Phi }_{L}^{h,\;A/Z}$) is the transport SBH of the electron (hole) in the lateral direction by LDDOS calculation (A and Z represent armchair and zigzag directions, respectively). $L_{g}^{A/Z}$ is the channel length of ML GeAs FET.

	Graphene	Ti_2_CF_2_	Ti_3_C_2_O_2_	V_2_CF_2_	Cu
$\bar{\varepsilon }$ (%)	2.36	3.04	3.18	3.09	0.32
*d*_z_ (Å)	3.28	2.67	2.58	2.63	2.04
*E*_b_ (eV/atom)	0.39	0.99	0.91	0.97	2.96
*W* (eV)	4.70	4.96	5.60	5.26	4.54
*W*_m_ (eV)	4.36	4.92	5.93	5.26	4.66
$\Phi_{V}$ (eV)	0.33	0.15	0	0	0
$\Phi_{L}^{e,\;Z}$ (eV)	1.52	1.18	1.18	1.42	0.57
$\Phi_{L}^{h,\;Z}$ (eV)	0.11	0.21	0.20	0.00	0.72
$\Phi_{L}^{e,\;A}$ (eV)	1.49	1.32	1.30	1.42	0.64
$\Phi_{L}^{h,\;A}$ (eV)	0.15	0.29	0.12	0.00	0.96
$L_{g}^{A}$ (Å)	68.1	67.1	67.1	67.1	66.8
$L_{g}^{Z}$ (Å)	54.7	53.7	53.8	53.6	54.7

## Data Availability

Data openly available in a public repository.

## Supplementary Materials

The following supporting information can be downloaded at: https://www.mdpi.com/article/10.3390/molecules28237806/s1, Table S1: Lattice constants of ML GeAs and independent 2D metal electrodes (graphene, Ti_2_CF_2_, V_2_CF_2_, and Ti_3_C_2_O_2_) and the Cu metal electrode.
