# Supplementary material for: Isotropic Contact Properties in Monolayer GeAs Field-Effect Transistors
Source: Molecules. 2023 Nov 27;28(23):7806. doi: 10.3390/molecules28237806 (PMC10708508; doi:10.3390/molecules28237806)
Supplement: Supplementary file 1 [file molecules-28-07806-s001.zip › molecules-2717705-supplementary.pdf]

Isotropic Contact Properties in Monolayer GeAs Field-Effect Transistors

Weiqi Song [1], Haosong Liu [2], Feihu Zou [1], Yize Niu [1], Yue Zhao [1], Yao Cong [2], Yuanyuan Pan [1,*] and Qiang Li [1,*]

[1] College of Physics, Center for Marine Observation and Communications, Qingdao University, Qingdao 266071, China; 2021020281@qdu.edu.cn (W.S.); 2021020280@qdu.edu.cn (F.Z.); 2022025400@qdu.edu.cn (Y.N.); zhaoyue69@qdu.edu.cn (Y.Z.)

[2] State Key Laboratory of Heavy Oil Processing, Institute of New Energy, College of Chemical Engineering, China University of Petroleum (East China), Qingdao 266580, China; lhs_upc@163.com (H.L.); cong_y1217@163.com (Y.C.)

* Correspondence: panyy@qdu.edu.cn (Y.P.); liqiang@qdu.edu.cn (Q.L.)

Table S1. Lattice constants of ML GeAs and independent 2D metal electrodes (graphene, Ti_2CF_2, V_2CF_2, and $Ti_3C_2O_2$) and the Cu metal electrode.

	**ML GeAs**	**graphene**	**Ti_2CF_2**	**V_2CF_2**	**$Ti_3C_2O_2$**	**Cu**
a (Å)	22.25	2.46	3.04	2.99	3.04	2.55
b (Å)	3.82	2.46	2.63	2.99	2.68	2.55
